# Sotalol Treatment may Interfere With Retrieval, Expression, and/or Reconsolidation Processes Thus Disrupting Traumatic Memories in a Post-Traumatic Stress Disorder Mice Model

**DOI:** 10.3389/fphar.2021.809271

**Published:** 2022-01-31

**Authors:** Raquel Martinho, Rafaela Seixas, Márcia Azevedo, Ana Oliveira, Paula Serrão, Mónica Moreira-Rodrigues

**Affiliations:** ^1^ Laboratory of Physiology, ICBAS - Institute of Biomedical Sciences Abel Salazar, University of Porto, Porto, Portugal; ^2^ Center for Drug Discovery and Innovative Medicines, ICBAS, University of Porto (MedInUP), Porto, Portugal; ^3^ Department of Biomedicine, FMUP - Faculty of Medicine, University of Porto, Porto, Portugal

**Keywords:** post-traumatic stress disorder, contextual traumatic memory, sotalol, β-adrenoceptors, peripheral β-adrenoceptor antagonist

## Abstract

The processes by which fear memory is encoded, consolidated, and re-consolidated are extremely complex and appear to require the release of stress hormones, especially adrenaline (AD). AD improves contextual fear memory, acting specifically on peripheral β2-adrenoceptors. Propranolol (peripheral and central β-adrenoceptor antagonist) treatment was shown to prevent post-traumatic stress disorder (PTSD) development and reduce its symptoms. However, propranolol has several side effects. Thus, we aimed to evaluate if sotalol (a peripheral β-adrenoceptor antagonist) treatment interferes with retrieval, expression, and/or reconsolidation of traumatic memories in a validated mice model that mimics the signs/symptoms of PTSD, thus intending to decrease them. Female mice were induced with PTSD following an established protocol. Sotalol (2.0 mg/kg) or vehicle were administered on days 2, 7, and 14. The percentage of freezing was calculated, and behavioral tests were carried out. Catecholamines in plasma were quantified by HPLC with electrochemical detection. Quantitative real-time polymerase chain reaction (qPCR) was used to evaluate mRNA expression of NR4A family genes in hippocampus. Following the submission of the animals to the same aversive context on days 2, 7, and 14, sotalol-treated mice exhibited significant less freezing behavior. In the elevated plus-maze test, the time spent and number of entries in the open arms, and total arm entries were increased in sotalol-treated mice. Also, the light-dark transition test revealed higher time spent, number of transitions to the light, and total number of transitions in sotalol-treated mice. Moreover, plasma AD was significantly decreased in sotalol-treated mice. On day 14, sotalol-treated mice exhibited a decrease in mRNA expression of *Nr4a1* in the hippocampus. In conclusion, in PTSD mice model, sotalol appears to decrease traumatic memories and anxiety-like behavior, probably due to a decrease in peripheral adrenergic activity, which influences traumatic memories. The effects of sotalol upon re-exposure to the traumatic context may be consistent with interference in the retrieval, expression, and/or reconsolidation processes of contextual traumatic memory, resulting in a long-term reduction of PTSD symptoms and signs. The decreased *Nr4a1* mRNA expression in the hippocampal formation may be crucial for these mice to develop diminished traumatic contextual memories after sotalol therapy in PTSD.

## 1 Introduction

Stress is an important response to maintain homeostasis when a threat occurs (McEwen, 1998). However, when such responses are inaccurately activated and regulated it may lead to serious pathologies, such as post-traumatic stress disorder (PTSD) (VanElzakker et al., 2014). PTSD is a widespread mental disturbance, included in trauma- and stressor-related disorders, which commonly co-occurs with other mental health disorders, such as anxiety (Brady et al., 2000). PTSD evolves from exposure to significant traumatic, frightening, or stressful experiences (Bracha, 2004; Goswami et al., 2013; Huang et al., 2020; Cenkner et al., 2021). Usually, PTSD patients manifest three wide groups of symptoms such as re-experiencing, avoidance, and hyperarousal, and show deficiencies in the processes that lead to the elimination of memories related to intense fear (Lissek et al., 2005; Goswami et al., 2013; Inslicht et al., 2013). In addition, patients diagnosed with PTSD present higher levels of hormones related to stress in plasma, namely catecholamines, such as noradrenaline (NA) and adrenaline (AD) (Sherin and Nemeroff, 2011; Pan et al., 2018). Moreover, when faced with trauma-related contexts, these patients exhibit increased blood pressure as well as significant changes in pulse, and skin conductance when compared with controls. Accordingly, there are studies reporting autonomic nervous system’s hyperactivity in PTSD patients (Li et al., 2006; Sherin and Nemeroff, 2011).

It has been described that PTSD is expected to affect women two to three times more than men (Olff, 2017). The higher predominance of PTSD in female patients could be attributable to variables that are not trauma-related, for instance, stress hormone sensitization in response to premature adverse circumstances, subjective perception of the incident, and intrinsic neuroendocrine factors (Seedat et al., 2005). Female PTSD patients may have greater symptoms, a longer illness course, and a poorer life quality than male patients (Seedat et al., 2005).

PTSD research is highly dependent on several animal models that have originated from the use of various forms of trauma experiences (Pynoos et al., 1996). The PTSD mice model chosen to be carried out in the present study is based on the concept that submission to electrical shocks leads the animals to develop PTSD’s pathophysiological processes and earliest complex symptoms and signs of the pathology, such as enhanced contextual traumatic memory and anxiety-like behavior (Li et al., 2006; Zhang et al., 2012; Martinho et al., 2020; Martinho et al., 2021). Previously, we and others reported that the exposure to multiple foot shocks is efficient to replicate the traumatic episode in this model (Li et al., 2006; Zhang et al., 2012; Verma et al., 2016; Martinho et al., 2020). Here, contextual cues appear to parallel the primary traumatic exposure of the stressful aversive situation. This is supposed to cause traumatic event re-experience (Gisquet-Verrier et al., 2004), replicating some of the characteristics found in PTSD patients. We have formerly reported that AD is increased in this PTSD mice model, leading to long-lasting contextual traumatic memories and anxiety-like behavior (Martinho et al., 2020).

Despite the relation between AD and contextual traumatic memories’ persistence (Alves et al., 2016; Oliveira et al., 2018; Martinho et al., 2020; Martinho et al., 2021), AD does not pass the blood-brain barrier and is known to have mainly peripheral actions. There are two theories concerning the role of this hormone in fear memory enhancement. The first theory mentions that AD can activate the glycogenolysis or gluconeogenesis in hepatocyte cells in the liver leading to blood glucose increase (Gray et al., 1980; Dufour et al., 2009), which in turn may be a source of energy for the synthesis and release of neurotransmitters. This may enhance contextual fear conditioning memory (Alves et al., 2016; Oliveira et al., 2018; Martinho et al., 2021). Another theory is that AD binds to the β-adrenoceptors of the ascending fibers of the vagus nerve. The peripheral terminals of the vagus innervate several structures that are affected by AD release by the adrenal gland, such as the heart or liver. The fibers of the vagus nerve then transmit the information regarding changes in those organs to the brainstem, namely to the nucleus of the solitary tract (NTS). Axonal terminal of the NTS neurons innervate and release neurotransmitters (namely NA) in the amygdala and hippocampus (Miyashita and Williams, 2006; Chen and Williams, 2012).

Propranolol (both peripheral and central β-adrenoceptor antagonist) treatment appears to both prevent the development and reduce PTSD symptoms (Pitman et al., 2002; Brunet et al., 2008). Furthermore, systemic administration of propranolol disrupted consolidation and reconsolidation of traumatic memories (Muravieva and Alberini, 2010). However, propranolol administration is related to several side effects (tiredness, slower heart rate, sleep, and gastrointestinal disturbances…) (Coté et al., 2018). Moreover, propranolol decreases memory consolidation also in non-aversive tasks and weakens memory reconsolidation in least aversive tasks (Villain et al., 2016). Therefore, the use of a peripheral β-adrenoceptor antagonist might be an alternative with fewer central side effects if it is shown to be effective. Considering the available literature, it is of our understanding that there are no reported studies that use sotalol as a treatment for PTSD in mouse models. The goal of the present research was to assess if treatment with sotalol interferes with the processes that lead to long-lasting traumatic memories in a PTSD mice model. This procedure has the potential to become a novel treatment approach for PTSD patients.

## 2 Material and Methods

### 2.1 Animals

Mice (129 × 1/SvJ) were bred at our animal facility where they were supervised under specific environmental conditions (12 h light/dark cycle, humidity 50%, room temperature 23 ± 1°C, autoclaved drinking water, *ad libitum* mice diet 4RF21/A; Mucedola, Milan, Italy) in cages with two to five animals. Adult female subjects (*n* = 26) with 8–12 weeks of age were randomly selected. Mice experienced a period of 12 h of light (8 a.m.–8 p.m.; light phase) during which the behavioral testing and physiological measurements were conducted, between 9 a.m. and 1 p.m. Previous studies reported that female rodents group-housed synchronize their ovarian cycles (McClintock, 1978). All experimental protocols involving animals were executed in agreement with European Directive number 63/2021/EU which translates to the Portuguese legislative Directive Law 113/2012 and 1/2019. Animal care and experiments were certified by the National Authority for Animal Health (DGAV) and the Organism Responsible for Animal Welfare in Faculty of Medicine of University of Porto.

### 2.2 PTSD Mice Model

**FIGURE 1:**
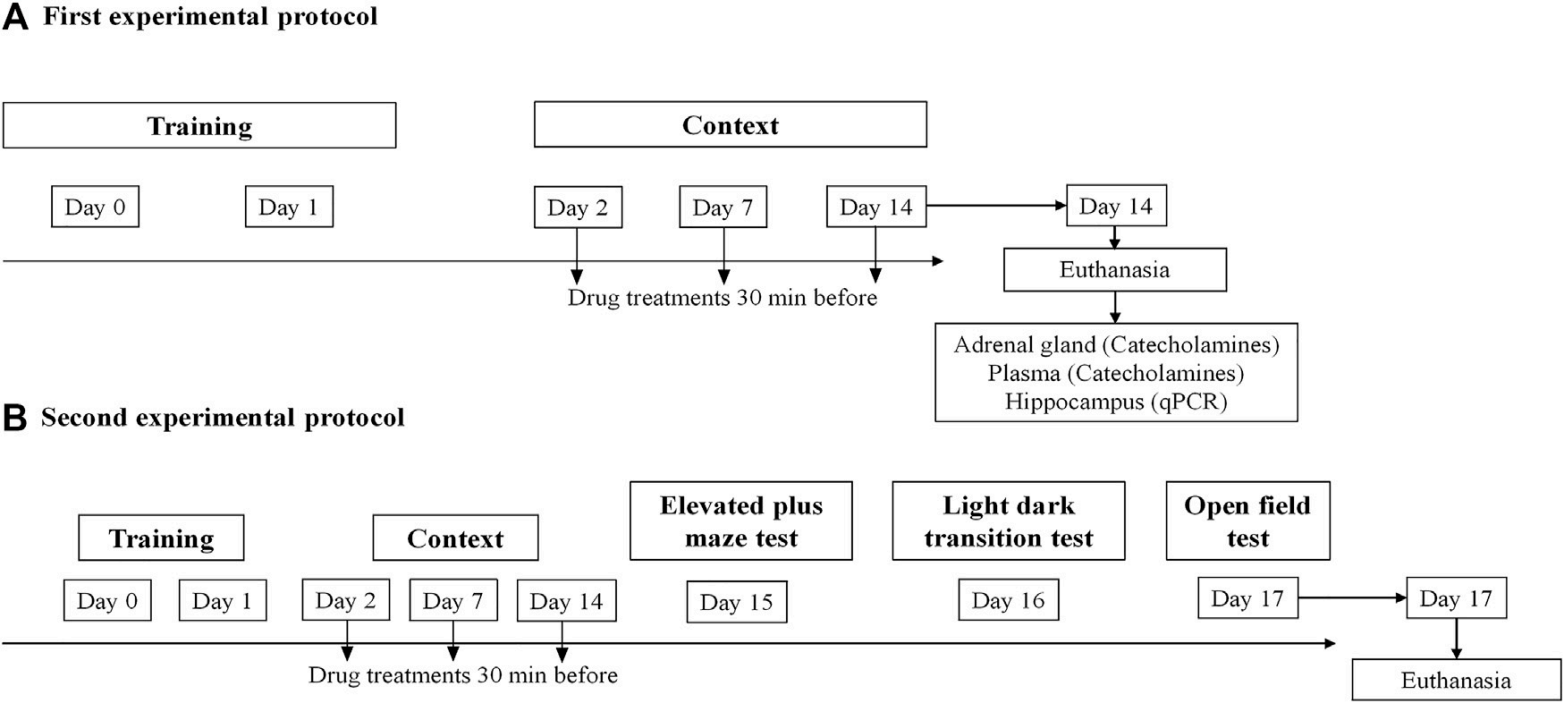
Schematic representation of the experimental design: treatments, behavioral protocols, and samples collection. **(A)** First experimental protocol and **(B)** second experimental protocol.

The mice model for the induction of PTSD was carried out similarly to previous studies (Pynoos et al., 1996; Li et al., 2006; Zhang et al., 2012; Verma et al., 2016; Martinho et al., 2020; Martinho et al., 2021). The material used for the training periods consisted of a transparent Plexiglass box with an electrified grid floor. On the first 2 days of the experimental protocol (days 0 and 1), all mice were subjected to an unpleasant event comprising 15 electric shocks (intensity, 0.8 mA; duration, 10 s; interval, 10 s) preceded by a 2-min habituation period. The training sessions lasted for a total of 7 min. Following the training period, the animals were re-exposed to the aversive context on days 2, 7, and 14. Re-exposure involved placing the animals in the same conditioned chamber without subjecting them to the foot shocks, for a total of 8 min. The induction and context chamber had a timer on top and the entire duration of the procedure was video recorded, allowing the posterior evaluation of freezing time. Freezing is defined as the lack of physical movements excluding those related to respiratory movements, for at least 3 s (Valentinuzzi et al., 1998), and was manually scored. The total number of audible vocalizations following a foot shock was also quantified when the mice produced a high-pitched squeak (Rocinholi et al., 1997). The behavioral tests were digitally recorded with a video camera Sony HDR-CX405 (Sony Corporation, Japan) and all analyzes were conducted under manual and blind protocols (Figure 1).

### 2.3 Drug Treatments

The experimental design consisted of two protocols. In both protocols, mice were administered intraperitoneally (i.p.) with sotalol [2 mg/kg, (Lee et al., 2001; Alves et al., 2016)] (*n* = 13) or vehicle (0.9% NaCl) (*n* = 13) 30 min before each session on days 2, 7, and 14. At the end of the first protocol (day 14), animals were administered with anesthetic (ketamine, 100 mg/kg and xylazine, 10 mg/kg; i.p.), for the collection of blood adrenal gland and hippocampus (Figure 1A). Following the second protocol, the animals underwent behavioral tests which were conducted sequentially on days 15, 16, and 17: elevated plus-maze test (EPMT), light-dark transition test (LDTT), and open field test (OFT), respectively (Figure 1B). Figure 1 is a schematic representation of the experimental design’s timeline, including behavioral protocols, treatments, and sample collections.

### 2.4 Behavioral Tests

#### 2.4.1 Elevated Plus-Maze Test

On day 15, the EPMT was performed (Figure 1B), as formerly reported (Pellow et al., 1985; Fertan et al., 2019; Martinho et al., 2020; Gasparyan et al., 2021; Martinho et al., 2021). The experimental device was positioned 60 cm above the floor and comprised two open arms (40 × 10 cm) and two enclosed arms (40 × 10 cm with 20 cm high walls), displaying a total of four arms delimiting a central area of 5 cm^2^ and arranged at right angles with equal arms facing each other. Initially, each mouse was gently positioned in the center of the device with its head facing a closed arm. A digital video camera Sony HDR-CX405 (Sony Corporation, Japan) was placed above the device and recorded each animal’s behavior for a total of 5 min. Resulting videos were analyzed manually and blinded, quantifying the time spent in open and closed arms, open arm entries, and total arm entries. The mice were considered to have made an entrance into an arm when all four paws were positioned into the novel compartment (Li et al., 2006; Zhang et al., 2012).

#### 2.4.2 Light-Dark Transition Test

On day 16, the LDTT was conducted (Figure 1B), as formerly defined (Li et al., 2006; Martinho et al., 2020; Silvestri et al., 2020; Martinho et al., 2021). The apparatus consisted of a wooden box without a lid comprising a light (50 × 30) and a dark compartment (50 × 20) with 25 cm high walls. The different areas were joined by a square entrance (10 × 10 cm), which remained open during the entire duration of the test. The test started by placing each animal into the light area facing the opposite wall to the entrance. A Sony digital video camera (Sony Corporation, Japan) was positioned above the apparatus and used to record the mice’s behavior, namely the time spent in the light area, the number of passages to the light area, and the total number of passages between dark and light areas. The test lasted for a total of 5 min, and results were obtained from manual and blind analyzes of the resulting videos.

#### 2.4.3 Open Field Test

On day 17, the OFT was carried out (Figure 1B), following previous reported protocols (Pynoos et al., 1996; Martinho et al., 2020; Martinho et al., 2021; Vidal et al., 2021). The test’s apparatus consisted of an open light wooden box (50 × 50) with 30 cm high walls. The chamber was light-colored with a square in the center (25 × 25 cm) and twelve squares on the periphery (12.5 × 12.5 cm) delimited on the floor with black lines. Initially, the animals were placed one at a time in the corner of the arena and left to freely explore the chamber for 10 min. A digital Sony HDR-CX405 video camera (Sony Corporation, Japan) was utilized to evaluate the behavioral aspects: number of squares crossed and entries in the central area. The results were obtained with a manual and blind analyses. The total distance travelled was video recorded and analyzed with an automated image analysis software (open-source software freely available at https://sourceforge.net/projects/toxtrac/) (Rodriguez et al., 2017; Rodriguez et al., 2018; Henry et al., 2019).

### 2.5 Quantification of Catecholamines

Following the first protocol, animals were administered with anesthetic (ketamine, 100 mg/kg and xylazine, 10 mg/kg; i.p.) on day 14 (Figure 1A) for sample collection. Left ventricle puncture was performed to collect blood to a heparinized tube. The blood was then placed in the centrifuge. The resulting plasma was frozen at −80°C pending further use. The alumina method was applied to concentrate the catecholamines present in plasma, as prior documented (Moreira-Rodrigues et al., 2014; Martinho et al., 2021). Electrochemical detection (mobile phase: citric acid 100mM, sodium acetate 100mM, 1-Octanesulfonic acid sodium salt 1.61mM, EDTA 0.15mM, Di-n-butylamine 1mM, methanol 10%; potential: 0.75 V) with limits of detection and quantification between 350 and 1,000 fmol and 700 and 2,000 fmol, respectively, was used to quantify the catecholamines, following the injection of 50 µL of plasma samples for separation by reverse-phase high-performance liquid chromatography (HPLC). Retention times were as follows: NA = 6.60 min, AD = 7.81 min, DA = 15.95 min.

### 2.6 RNA Isolation and Relative Quantification of mRNA Expression

Hippocampus samples were collected on day 14 and submitted to the quantitative real-time PCR (qPCR) protocol (Figure 1A), as described before (Moreira-Rodrigues et al., 2007; Mendes et al., 2018; Oliveira et al., 2018; Martinho et al., 2020). Total RNA isolation was performed using the Mini Kit illustra™ Isolate II RNA (Bioline, London, United Kingdom). NanoDrop 2000 spectrophotometer (Thermo Scientific, Waltham, United States) was utilized to measure the concentration and purity of the isolated RNA. A T100™ Thermal Cycler (Bio-Rad, Hercules, United States) was used to carry out reverse transcription, applying a Reverse Transcription kit (NZY First-Strand cDNA Synthesis Kit NZYTech—Genes and Enzymes, Lisbon, Portugal). StepOne™ real-time PCR System (Applied BioSystems, Waltham, United States) allowed the conduction of qPCR reactions. Gene-specific primers (10 µM), Maxima SYBR Green qPCR Master Mix (Thermo Scientific, Waltham, MA, United States), RNase-free H_2_O (Bioline, London, United Kingdom) were mixed and cDNA was added (1:20). Instead of cDNA, RNase-free H_2_O (Bioline, London, United Kingdom) was added as a negative control. Gene-specific primers are presented in Table 1. Results of mRNA quantification were expressed in an arbitrary unit (AU) after normalization for Glyceraldehyde 3-phosphate dehydrogenase (GAPDH).

**TABLE 1:** Primers used in gene expression analysis.

Gene	Primer (5’→ 3’)
*Nr4a1*	F: AAA​ATC​CCT​GGC​TTC​ATT​GAG
	R: TTT​AGA​TCG​GTA​TGC​CAG​GCG
*Nr4a2*	F: CGG​TTT​CAG​AAG​TGC​CTA​GC
	R: TTG​CCT​GGA​ACC​TGG​AAT​AG
*Nr4a3*	F: GTG​GCT​CGA​CTC​CAT​TAA​AGA​C
	R: GTG​CAT​AGC​TCC​TCC​ACT​CTC​T
*Gapdh*	F: CCA​TCA​CCA​TCT​TCC​AGG​AG
	R: GCA​TGG​ACT​GTG​GTC​ATG​AG

*Nr4*, Nuclear receptor 4; *Gapdh*, Glyceraldehyde 3-phosphate dehydrogenase; F, forward primer and R, reverse primer.

### 2.7 Drugs

(±)-Sotalol hydrochloride, (-)-adrenaline, L-(-)-noradrenaline, dopamine hydrochloride, 2,3-dihydroxybenzoic acid, and perchloric acid were purchased from Sigma-Aldrich (St. Louis, United States). Ketamine (Imalgene 1,000, Merial, Lisboa, Portugal) and xylazine (Rompum 2%, Bayer, Lisboa, Portugal) were purchased from Agrofauna (Vila Nova de Gaia, Portugal).

### 2.8 Statistics

The minimum number of animals required for the experimental protocols was determined by an online Sample Size Calculator (https://clincalc.com/Stats/SampleSize.aspx). Obtained data was displayed as means ± standard error of the means (SEM) and the statistical analyses were performed using the GraphPad Prism 6 (GraphPad Software Inc., La Jolla, United States). Results regarding freezing behavior were analyzed by Two-Way Analysis of Variance (ANOVA) repeated measures, followed by Sidak’s *post-hoc* test using treatment as “between-subjects factor” and time as “within-subjects factor” (repeated measure). Student’s *t*-test was applied to data concerning other behavioral results, catecholamines concentration, and qPCR. The presence of outliers was evaluated using GraphPad Prism 6. For all analyzes, significance level was set at 0.05.

## 3 Results

### 3.1 Sotalol-Treated PTSD Mice Have Decreased Contextual Traumatic Memory

**FIGURE 2:**
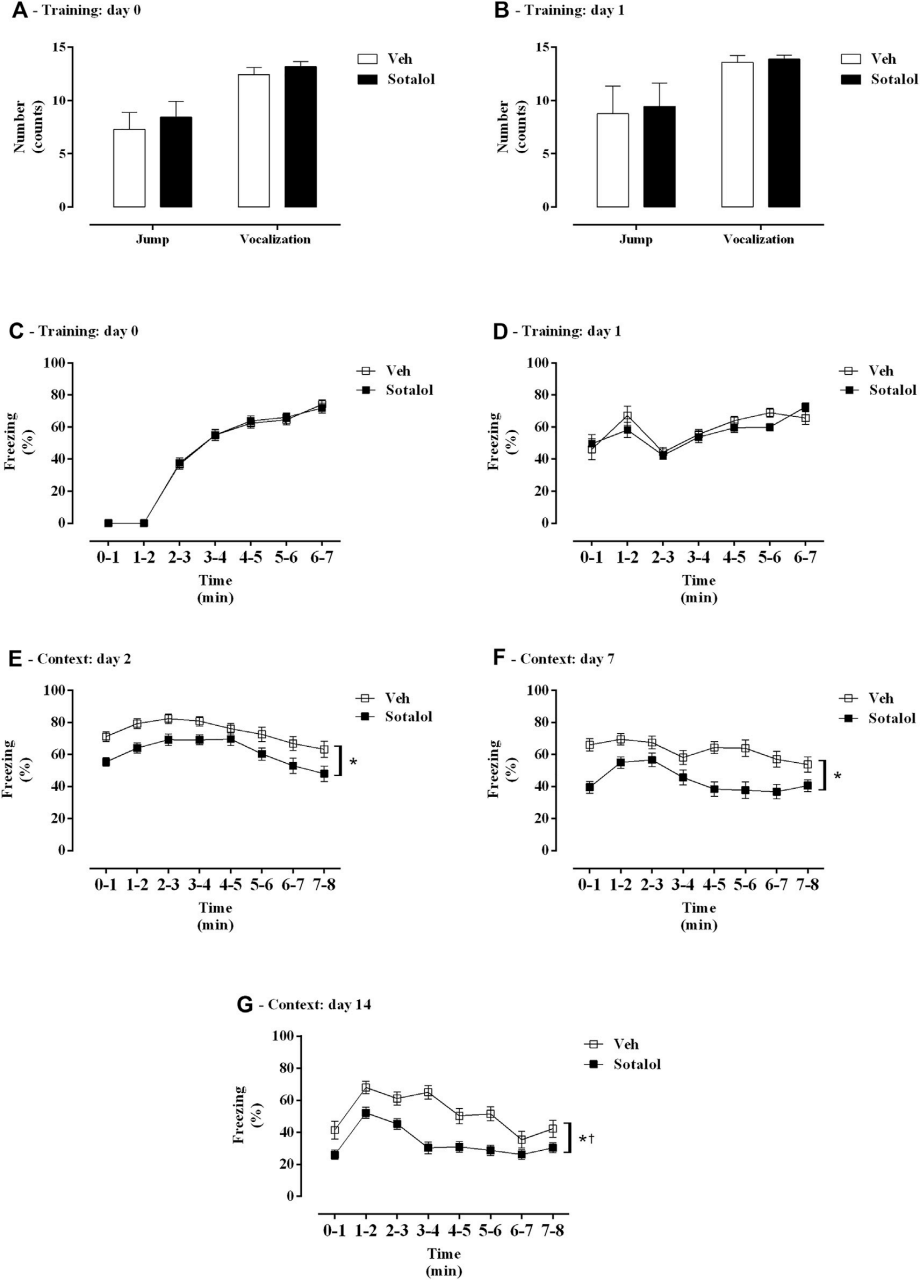
**(A, B)** Shock responsivity and **(C–G)** freezing behavior during induction of post-traumatic stress disorder (PTSD) on **(A, C)** day 0, **(B, D)** day 1, **(E)** day 2, **(F)** day 7, and **(G)** day 14. Veh, vehicle-treated PTSD mice; Sotalol, sotalol-treated PTSD mice. Values are means ± SEM of 13 mice per group. *, treatment and time effect with two-way repeated measures ANOVA (*p* < 0.05). †, interaction with two-way repeated measures ANOVA (*p* < 0.05).

Figure 2 represents the outcome of treatment with sotalol in freezing, vocalization and jump behavior of PTSD mice. Animals in different groups exhibited no statistical differences in jump (t _(26)_ = 1.01, *p* = 0.3, Figure 2A; t _(26)_ = 0.16, *p* = 0.9, Figure 2B), vocalization (t _(26)_ = 1.26, *p* = 0.2, Figure 2A; t _(26)_ = 0.80, *p* = 0.4, Figure 2B), or freezing responses (F _(1, 26)_ = 0.49, *p* = 0.5, Figure 2C; F _(1, 26)_ = 1.96, *p* = 0.2, Figure 2D) during training days (0 and 1). A significant effect of time (F _(6, 252)_ = 306.40, *p* < 0.0001, Figure 2A; F _(6, 252)_ = 12.17, *p* < 0.0001, Figure 2B; F _(1, 84)_ = 18.46, *p* < 0.0001, Figure 2C; F _(1, 84)_ = 7.64, *p* = 0.007, Figure 2D) was observed. No significant effect of treatment (F _(1, 42)_ = 0.02, *p* = 0.9, Figure 2A; F _(1, 42)_ = 0.83, *p* = 0.4, Figure 2B; F _(1, 84)_ = 0.68, *p* = 0.4, Figure 2C; F _(1, 84)_ = 0.09, *p* = 0.8, Figure 2D) or interaction (F _(6, 252)_ = 0.1, *p* = 0.9, Figure 2A; F _(6, 252)_ = 1.22, *p* = 0.1, Figure 2B; F _(1, 84)_ = 0.03, *p* = 0.9, Figure 2C; F _(1, 84)_ = 0.01, *p* = 0.9, Figure 2D) were present.

Also, on context days, days 2, 7 and 14, a significant effect of time (F _(7, 532)_ = 13.62, *p* < 0.0001, Figure 2E; F _(7, 532)_ = 5.82, *p* < 0.0001, Figure 2F; F _(7, 532)_ = 19.10, *p* < 0.0001, Figure 2G) and treatment (F _(1, 76)_ = 10.61, *p* = 0.002, Figure 2E; F _(1, 76)_ = 18.73, *p* < 0.0001, Figure 2F; F _(1, 76)_ = 27.45, *p* < 0.0001, Figure 2G) were observed. No significant interaction (F _(7, 532)_ = 0.56, *p* = 0.8, Figure 2E; F _(7, 532)_ = 1.86, *p* = 0.1, Figure 2F) was observed on days 2 and 7. A significant interaction was observed on day 14 (F _(7, 532)_ = 2.90, *p* = 0.006, Figure 2G).

### 3.2 Sotalol Treatment Decreased Anxiety-like Behavior in PTSD

**FIGURE 3:**
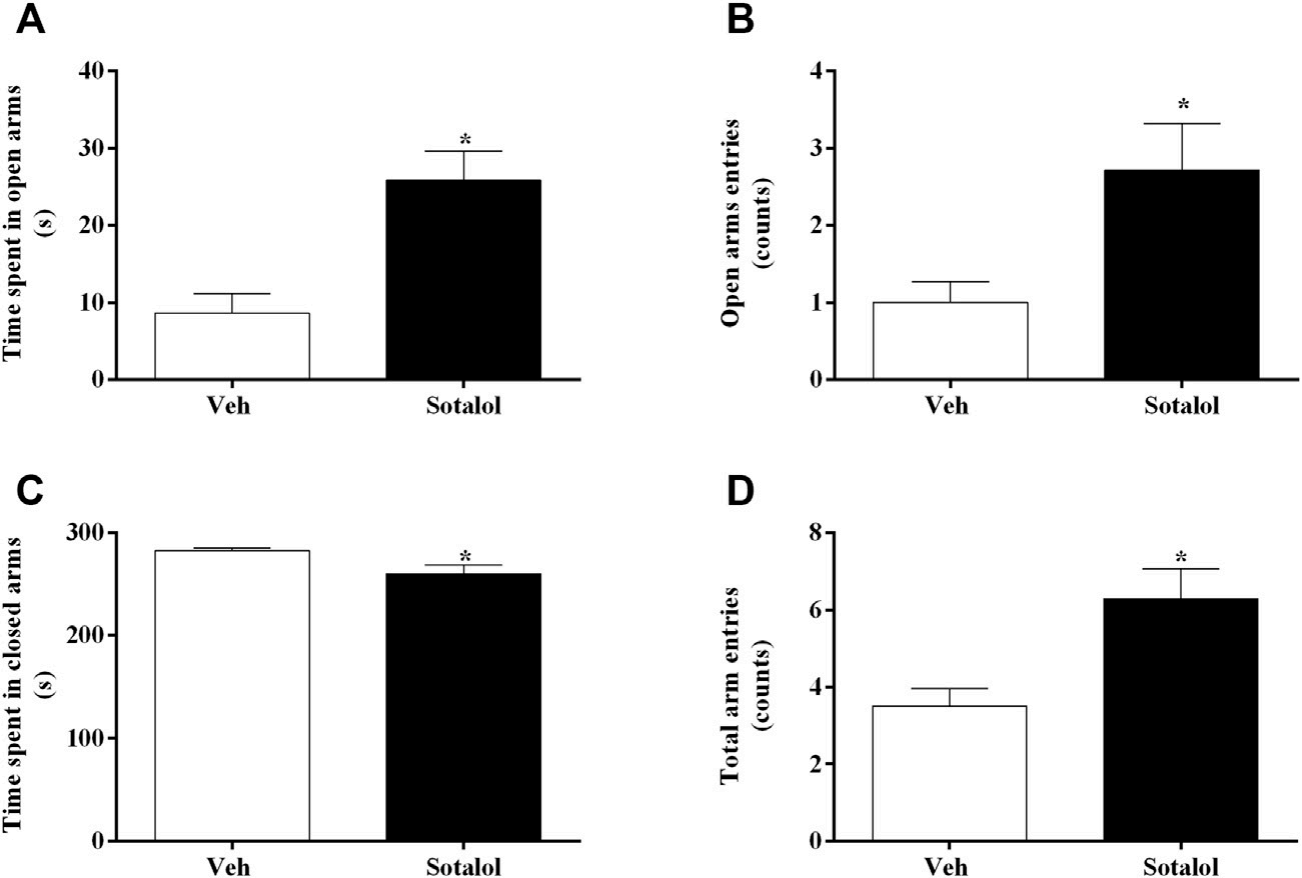
**(A)** Time spent in open arms, **(B)** open arm entries, **(C)** time spent in closed arms, and **(D)** total arm entries of the elevated plus-maze test (EPMT), on day 15 after post-traumatic stress disorder (PTSD) induction. Veh, vehicle-treated PTSD mice; Sotalol, sotalol-treated PTSD mice. Values are means ± SEM of 6-7 mice per group. *, significantly different from correspondent values in vehicle-treated PTSD mice (*p* ˂ 0.05).

To access the effects of sotalol treatment on anxiety-like behavior, the EPMT (day 15) and LDTT (day 16) were conducted. In the EPMT, sotalol-treated mice displayed higher time spent in open arms (t _13)_ = 3.9, *p* = 0.002, Figure 3A), open arm entries (t _13)_ = 2.7, *p* = 0.02, Figure 3B), and total number of arm entries (t _13)_ = 3.2, *p* = 0.007, Figure 3D). Accordingly, mice administered with sotalol also presented less time occupying closed arms (t _13)_ = 2.6, *p* = 0.02, Figure 3C) in comparison with vehicle-treated mice.

**FIGURE 4:**
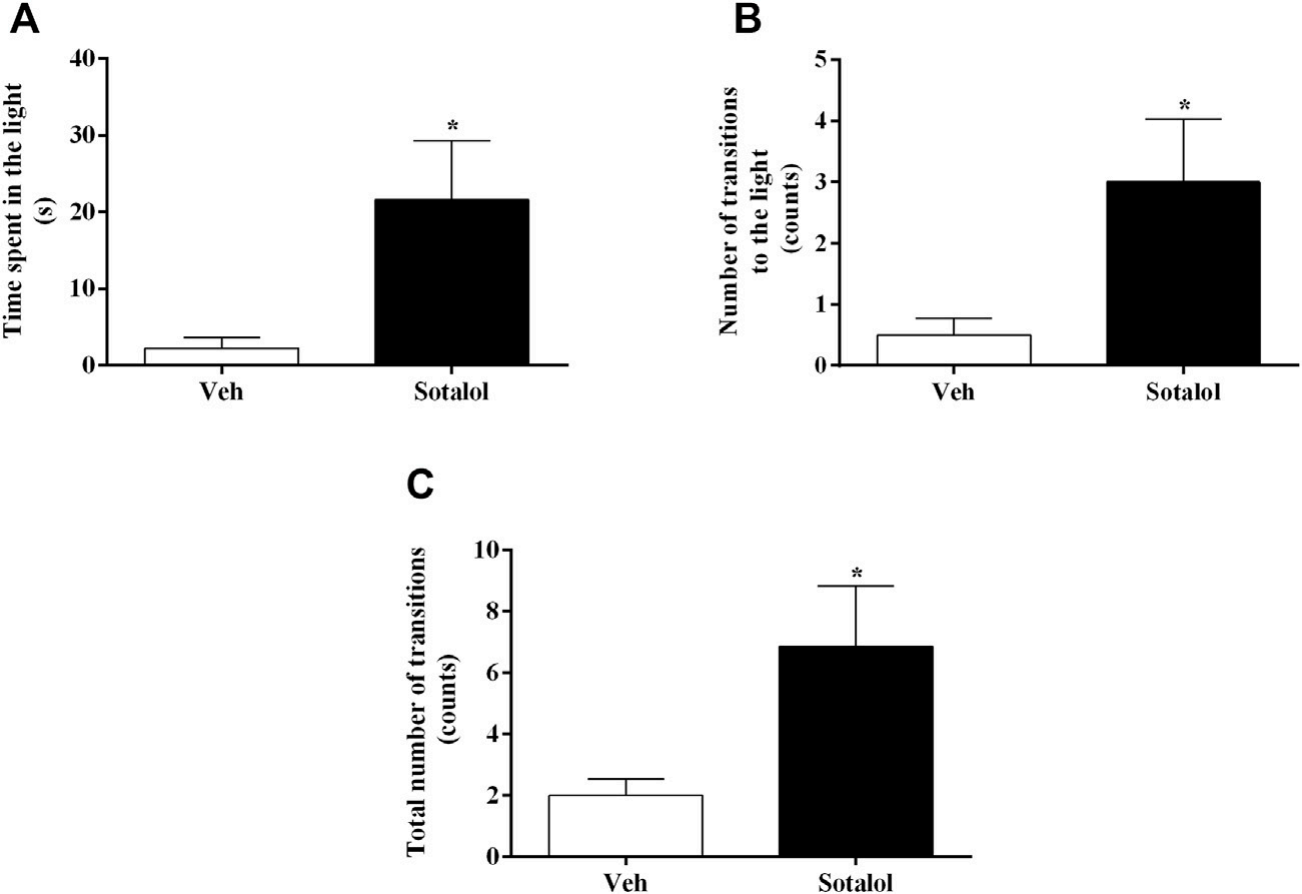
**(A)** Time spent in the light compartment, **(B)** number of transitions to the light compartment, and **(C)** total number of transitions between light and dark compartments of light-dark transition test (LDTT), on day 16 after post-traumatic stress disorder (PTSD) induction. Veh, vehicle-treated PTSD mice; Sotalol, sotalol-treated PTSD mice. Values are means ± SEM of 6-7 mice per group. *, significantly different from correspondent values in vehicle-treated PTSD mice (*p* ˂ 0.05).

In the LDTT, sotalol-treated mice showed a significant increase in the time spent in the light compartment (t _13)_ = 2.6, *p* = 0.02, Figure 4A), in the number of transitions to the light compartment (t _13)_ = 2.5, *p* = 0.02, Figure 4B), and in the total number of transitions between different areas (t _13)_ = 2.5, *p* = 0.02, Figure 4C), compared to vehicle-treated mice.

### 3.3 Sotalol Treatment Did not Affect Locomotor Activity

**FIGURE 5:**
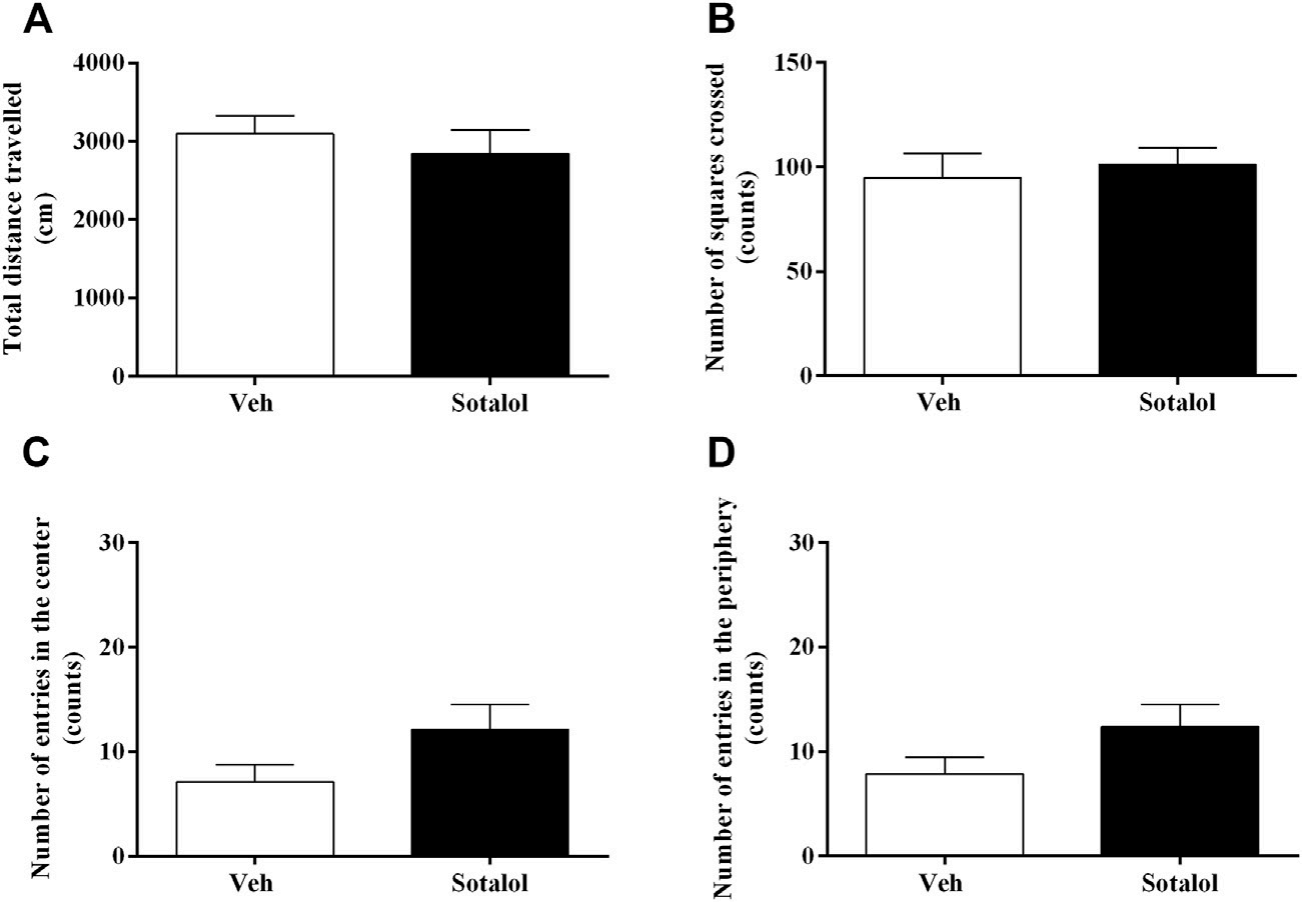
**(A)** Total distance travelled, and number of **(B)** squares crossed, **(C)** entries in the center, and **(D)** entries in the periphery in the open field test (OFT), on day 17 after post-traumatic stress disorder (PTSD) induction. Veh, vehicle-treated PTSD mice; Sotalol, sotalol-treated PTSD mice. Values are means ± SEM of 6-7 mice per group.

On day 17, the OFT was carried out to access the outcome of sotalol administration on locomotor activity. The sum of distance travelled (t _13)_ = 0.7, *p* = 0.5, Figure 5A), number of squares crossed (t _13)_ = 0.5, *p* = 0.6, Figure 5B), entries in the center (t _13)_ = 1.7, *p* = 0.1, Figure 5C), and entries in the periphery (t _13)_ = 1.7, *p* = 0.1, Figure 5D) were not significantly different between groups.

### 3.4 Sotalol Decreased Plasma AD in PTSD

**FIGURE 6:**
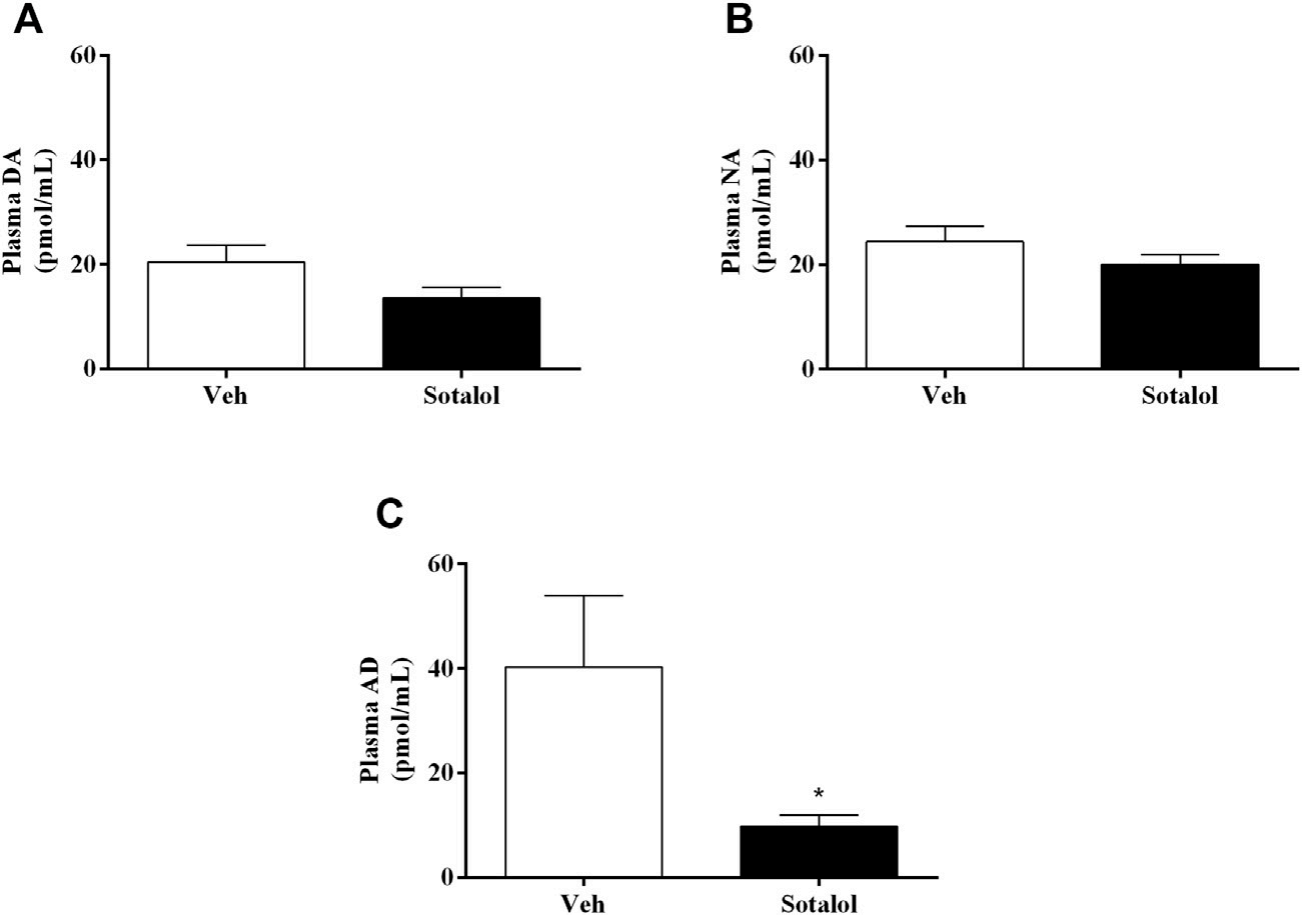
Concentration of plasma **(A)** dopamine (DA), **(B)** noradrenaline (NA), and **(C)** adrenaline (AD) after post-traumatic stress disorder (PTSD) induction (day 14). Values are means ± SEM of 6-7 mice per group. Veh, vehicle-treated PTSD mice; Sotalol, sotalol-treated PTSD mice. *, significantly different from correspondent values in vehicle-treated PTSD mice (*p* ˂ 0.05).

**FIGURE 7:**
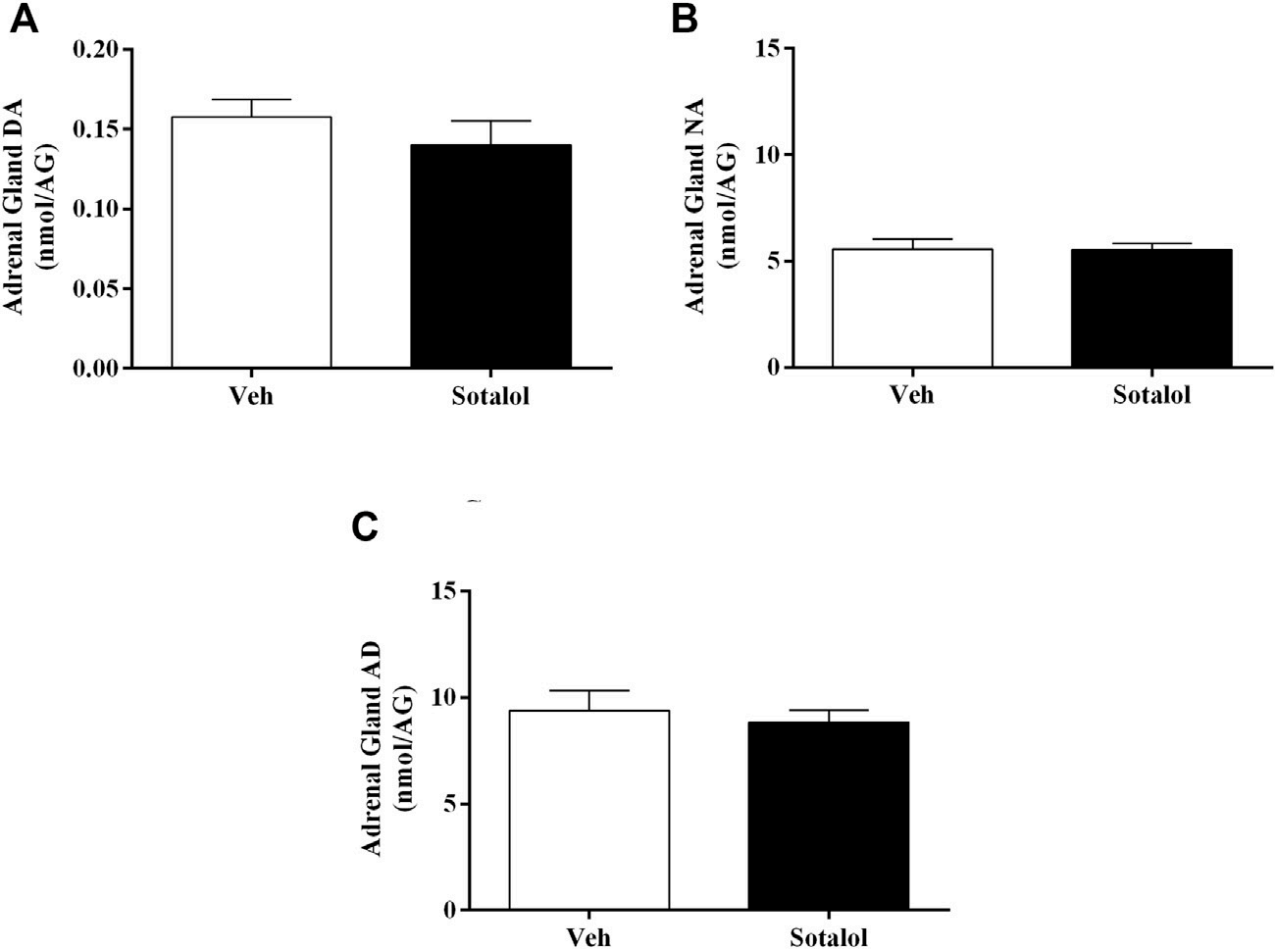
Concentration of adrenal gland **(A)** dopamine (DA), **(B)** noradrenaline (NA), and **(C)** adrenaline (AD) after post-traumatic stress disorder (PTSD) induction (day 14). Values are means ± SEM of 6-7 mice per group. Veh, vehicle-treated PTSD mice; Sotalol, sotalol-treated PTSD mice.

To determine the consequences of sotalol treatment in catecholamines, quantification levels in plasma and adrenal gland 14 days after PTSD induction was assessed. No statistical differences were observed in plasma DA (t _(13)_ = 1.6, *p* = 0.1, Figure 6A) and NA (t _(13)_ = 1.2, *p* = 0.2, Figure 6B) between sotalol-treated and vehicle-treated mice. Plasma AD (t _(13)_ = 2.2, *p* = 0.048, Figure 6C) was significantly decreased in sotalol-treated mice in comparison with vehicle-treated mice. Furthermore, there were no statistically significant differences in adrenal gland DA (t _(13)_ = 0.9, *p* = 0.4, Figure 7A), NA (t _(13)_ = 0.03, *p* = 0.9, Figure 7B) and AD (t _(13)_ = 0.5, *p* = 0.6, Figure 7C) between sotalol-treated and vehicle-treated mice.

### 3.5 Sotalol Decreased *Nr4a1* Gene Expression in the Hippocampus of Sotalol-Treated Mice

**FIGURE 8:**
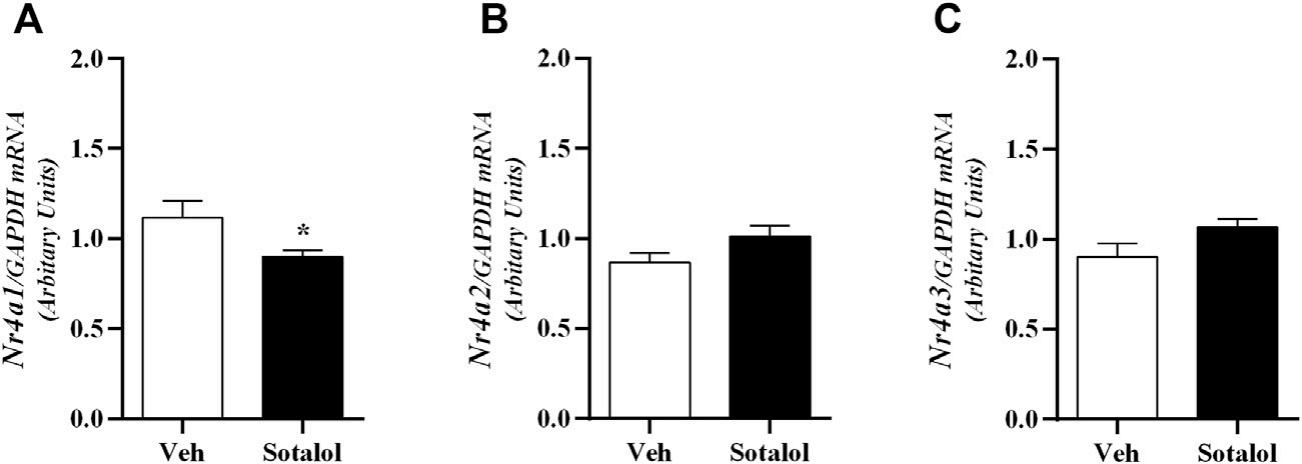
Hippocampus mRNA expression of **(A)** Nuclear receptor 4 (*Nr4*) *a1*; **(B)** *Nr4a2*, and **(C)** *Nr4a3* on day 14 of post-traumatic stress disorder (PTSD) induction. Values are means ± SEM of 6-7 mice per group. Results of mRNA are expressed as arbitrary units (AUs) after normalization for glyceraldehyde 3-phosphate dehydrogenase (GAPDH). Veh, vehicle-treated PTSD mice; Sotalol, sotalol-treated PTSD mice. *, significantly different from correspondent values in vehicle-treated PTSD mice (*p* ˂ 0.05).

Hippocampus mRNA expression of *Nr4a1* (t _(13)_ = 2.2, *p* = 0.04; Figure 8A) was significantly decreased in sotalol-treated mice when compared to mice treated with vehicle. Also, no statistical differences were observed in mRNA expression of *Nr4a2* (t _(13)_ = 1.8, *p* = 0.1; Figure 8B) and Nr4a3 (t _(13)_ = 1.9, *p* = 0.1; Figure 8C) between sotalol-treated and vehicle-treated mice 14 days after PTSD induction.

## 4 Discussion

In this study, the animals were subjected to a validated animal model of PTSD (Li et al., 2006; Zhang et al., 2012; Verma et al., 2016; Martinho et al., 2020). In this mouse model, several foot shocks have been reported to mimic the traumatic event that typically leads to the appearance of the symptoms and signs seen in patients diagnosed with PTSD and comorbid disorders, such as anxiety (Fanselow et al., 1993; Brady et al., 2000; Rau et al., 2005). Submitting the animals to the same context in which the aversive stimuli were delivered appears to replicate the effects of the traumatic event triggers experienced by patients with PTSD (Gisquet-Verrier et al., 2004; Verma et al., 2016; Martinho et al., 2020). Upon re-exposure to the contextual reminders, the animals present a freezing response which is a quantitative evaluation of conditioned associative fear memory and can represent the response to trauma-related signs and PTSD symptoms (Siegmund and Wotjak, 2007).

Cain et al. had previously shown that adrenergic activity is likely to play a role in fear extinction, by administration of two adrenoceptor antagonists (yohimbine and propranolol) in the fear conditioning test (Cain et al., 2004). Propranolol is both a peripheral and central β-adrenoceptor antagonist and appears to reduce PTSD symptoms (Pitman et al., 2002; Brunet et al., 2008). However, propranolol has significant secondary effects such as fatigue, bradycardia, sleep, and gastrointestinal disturbances... (Coté et al., 2018). In addition, propranolol decreases memory consolidation in non-aversive tasks and weakens memory reconsolidation in least aversive tasks (Villain et al., 2016). To our best understanding, there are no reported studies with a peripheral β-adrenoceptor antagonist treatment, such as sotalol, in PTSD mouse models.

In the present study, we report no differences in jump, vocalization, and freezing responses on days 0 and 1, suggesting that the traumatic event generated by foot shocks was consistent across both groups. Sotalol was administered 30 min before context days (days 2, 7 and 14), following induction of PTSD (day 0 and 1). After PTSD induction, we observed that the freezing behavior was decreased in sotalol-treated mice in comparison with vehicle-treated mice on days of re-exposure to contextual cues (days 2, 7, and 14). Accordingly, in the presented PTSD mice model, sotalol treatment may have interfered with retrieval, expression, and/or reconsolidation processes of traumatic contextual fear memories.

The elevated plus-maze test allows the evaluation of anxiety-like responses in rodents (Pellow et al., 1985) and is focused on the inherent propensity of animals to steer clear of open and elevated places. Additionally, it is also based on the animal’s innate exploratory behavior in novel environments (Zhang et al., 2012; Martinho et al., 2020). Findings in our study showed that sotalol-treated mice had increased time spent in open arms, number of entries into the open arms, and total arm entries when compared to vehicle-treated mice. The light-dark transition experiment focuses on the rodents’ natural aversion towards luminous areas and their natural exploratory behavior when moderate stressors, such as a new environment and light are applied (Bourin and Hascoet, 2003). Our findings showed an increase in the time spent in the light, the number of transitions to the light, and the total number of transitions between compartments in sotalol-treated mice in comparison with vehicle-treated mice. Thus, taking together the results, sotalol treatment seems to have reduced the anxiety-like behavior in PTSD-induced mice. The decrease in both contextual fear memory and anxiety-like behavior after experiencing a traumatic episode and being treated with sotalol made the mice more likely to develop a weaker traumatic contextual memory compared to those which weren’t treated. This may decrease the persistence of PTSD-associated symptoms. Sotalol may be a possible therapy for the reduction of PTSD symptoms and signs.

Preceding articles suggested that foot shocks linked to specific reminders of an event had no impact on the mice’s mobility associated with the open-field experiments carried out after PTSD mouse model induction (Pynoos et al., 1996; Martinho et al., 2020). Moreover, we evaluated if treatment with sotalol could exert effects on the mice’s locomotor activity. Our findings showed no differences in the evaluated parameters of the test, namely in total distance traveled, number of squares crossed, entries in the center and the periphery. Furthermore, total arm entries in the elevated plus-maze test do not represent an ideal indicator of locomotor activity (Walf and Frye, 2007), since there are other behavioral tendencies affecting the performance. Indeed, the higher number of arm entries observed after sotalol treatment was not linked with an analogous effect on locomotor activity, as proven by the open field test results. Thus, the behavioral changes that result from the sotalol treatment do not appear to be due to locomotor activity differences, but to behavioral predisposition to lower anxiety (Li et al., 2006; Zhang et al., 2012; Martinho et al., 2020). The OFT has also been thoroughly described as an alternative test for the evaluation of anxiety, less anxious mice tend to spend more time in the center of the arena. Nevertheless, studies showed that even though classical treatments such as benzodiazepine receptor full agonists elicit anxiolytic-like effect in the OFT in most cases (approximately 2/3), compounds such as serotonin reuptake inhibitors, commonly used for PTSD’s treatment (sertraline, paroxetine, etc.) were poorly effective as anxiolytics in the same test (Prut and Belzung, 2003; Gogas et al., 2007). Review of the literature suggest that the OF paradigm does not claim predictive validity for anxiety in general, as it is not sensitive to several compounds effective in anxiety disorder’s therapy.

It was previously suggested that catecholamines may be involved in the potentiation of emotional memories (Cahill et al., 1994). β-adrenoceptors activation influences long-term declarative memory consolidation for emotionally stressful events that trigger the release of adrenergic hormones (Cahill and McGaugh, 1998). Considering the vagal pathway, β-adrenoceptors on the vagus nerve may be activated by catecholamines released from the adrenal gland following a stressful event, causing glutamate to be released in the NTS neurons, activating them. Afterwards, glutamate is released in locus coeruleus neurons, inducing NA to be released in the hippocampus and basolateral amygdala (Miyashita and Williams, 2006; Noble et al., 2019). Contextual fear memory consolidation may be enhanced as a result of NA release in several cortical and subcortical regions. Moreover, stronger recollections of events could be preceded by peripheral sympathetic arousal (Fanselow, 2013). On the other hand, the liver glucose theory states that AD is released into the bloodstream in response to arousal, stimulating liver β_2_-adrenoceptors, which causes glycogenolysis, raising blood glucose which may cross the blood-brain barrier and influence memory (Morris and Gold, 2013). Accordingly, our group has previously shown that the release of AD may be especially important for the persistence of traumatic memories in PTSD (Martinho et al., 2020). Moreover, the observed traumatic contextual memory, anxiety-like behavior, and catecholamine levels in vehicle-treated PTSD mice presented in the current study, agree with previous reports from our group in which PTSD and non-PTSD mice were compared (Martinho et al., 2020).

The acute administration of sotalol (5 mg/kg) was shown to decrease plasma catecholamines concentration and chronic treatment with this drug causes a significant reduction in the sympathoadrenal basal activity (Bouvier and de Champlain, 1986). In agreement with this study, we observed that acute sotalol treatment decreased AD in plasma 14 days after PTSD induction. According to the theories mentioned above, sotalol decreases AD availability and its binding to the peripheral β-adrenoceptors may prevent AD to exert its action in the liver’s β_2_-adrenoceptors or the β-adrenoceptors on the vagus nerve, affecting glucose release by hepatocytes and/or activation of the nerve, respectively. Therefore, less glucose may be available as an energy source in astrocytes and neurons and may occur less adrenergic neuronal activation due to decreased NA release in the amygdala and hippocampus. This decrease may explain the behavioral changes observed in this mice model. Moreover, catecholamines released are firmly regulated by catecholamines themselves (Eisenhofer and Lenders, 2018), and so disrupting AD binding to β-adrenoceptors appears to result in a decreased release of adrenal AD in sotalol-treated PTSD mice on day 14, the third day of re-exposure to the context. All these effects may contribute to the observed decrease in contextual traumatic memories formation in this PTSD mice model with sotalol treatment. Our results suggest that inhibiting adrenergic activity during the process of retrieval, expression and/or reconsolidation may influence the persistence of traumatic memories in PTSD.

One limitation of the experimental design planned for this study is that it does not allow for disambiguation of retrieval and reconsolidation effects of sotalol, since the two appear to be inherently connected: reconsolidation may refer to the process through which retrieval of a memory after the initial consolidation causes it to become labile, and susceptible to modification (Auchter et al., 2017). Sotalol decreases catecholamines in plasma 20 min after acute administration (Rodriguez-Romaguera et al., 2009) and produces peak plasma levels for at least 2 h after treatment (Brown et al., 1976). Therefore, by the time the mice are re-exposed to the aversive context, 30 min after sotalol administration, the observed effects could be a result of disruption of either retrieval or reconsolidation.

Moreover, we opted to explore the hippocampal gene expression to better understand the molecular basis of the impairment of traumatic memories in PTSD when associated with a β-adrenoceptor antagonist treatment. The hippocampus appears to be implicated in the development of contextual fear memories, as well as in new gene transcription and protein synthesis to form long-term memories (Flexner et al., 1963; Phillips and LeDoux, 1992). An enhanced formation of contextual fear memories might be related to an increase of *Nr4a* family genes transcription, namely *Nr4a2* and *Nr4a3*, on the hippocampal formation (Siegmund and Wotjak, 2007). In agreement with these theories our group has suggested that glucose may be a mediator of AD in the central nervous system inducing the expression of the *Nr4a* family genes involved in contextual fear memories (Oliveira et al., 2018). This is in accordance with other publications which propose a role of these genes in the formation of long-term contextual fear memories (McQuown et al., 2011; Hawk et al., 2012). Consistently, contextual fear memory was increased when WT mice were administered with trichostatin A (TSA), a histone deacetylase (HDAC) inhibitor, which increases histone acetylation thus altering chromatin structure and increasing accessibility for transcriptional regulatory proteins. Consequently, TSA increases *Nr4a1*, *Nr4a2*, and *Nr4a3* expression in the hippocampus. Accordingly, these transcription factors appear to be essential for synaptic plasticity in the hippocampus (Hawk et al., 2012).

Our group previously observed that AD-deficient mice (Pnmt-KO) had lower *Nr4a* mRNA expression in the hippocampus compared to WT mice, and possibly a blunted glucose raise after a fear event, decreasing the energy source to the brain and thus decreasing contextual fear memory. In addition, *Nr4a* mRNA expression improved in AD-treated Pnmt-KO mice and was accompanied by enhanced contextual fear memory (Oliveira et al., 2018). Also, *Nr4a* mRNA expression was increased in the hippocampus in WT mice induced with PTSD in comparison with WT mice not induced with PTSD (Martinho et al., 2020). Accordingly, 14 days after PTSD induction, we observed a decrease in *Nr4a1* mRNA expression in the hippocampus of sotalol-treated PTSD mice accompanied by a decrease in traumatic memory. Therefore, since sotalol is a peripheral β-adrenoceptor antagonist it may prevent AD from acting in the central nervous system by blocking the binding of AD to β-adrenoceptors, and thus through interference with liver glucose and vagus nerve pathways previously described. And this may explain the decrease of *Nr4a1* mRNA expression in the hippocampus in sotalol-treated mice.

In conclusion, in this PTSD mouse model, sotalol decreases traumatic contextual memories and anxiety-like behavior, possibly due to a lack of action of AD in peripheral β-adrenoceptors, leading to a decrease in peripheral adrenergic activity, which influences the traumatic memory in the brain. Re-exposure to the traumatic context combined with the disruptive effects of sotalol affected contextual traumatic memory retrieval, expression, and/or reconsolidation. The decrease in *Nr4a1* mRNA expression in the hippocampus may be important to develop a weaker traumatic contextual memory after sotalol treatment. Therefore, peripheral β-adrenoceptor antagonist sotalol may be a possible treatment for PTSD.

## Data Availability

The original contributions presented in the study are included in the article/Supplementary Material, further inquiries can be directed to the corresponding author.
